# Reduced task-induced frontal midline theta activity in chronic stroke patients compared to healthy older adults – An MEG study

**DOI:** 10.1016/j.nicl.2026.103984

**Published:** 2026-03-06

**Authors:** Rebekah I. Brückner, Jale Özyurt, Christiane M. Thiel, Christoph S. Herrmann, Florian H. Kasten

**Affiliations:** a Biological Psychology Lab, Department of Psychology, School of Medicine and Health Sciences, Carl von Ossietzky Universität Oldenburg, Oldenburg, Germany; b Research Center Neurosensory Science, Carl von Ossietzky Universität Oldenburg, Oldenburg, Germany; c Experimental Psychology Lab, Department of Psychology, School of Medicine and Health Sciences, Carl von Ossietzky Universität Oldenburg, Oldenburg, Germany; d Department for Cognitive, Affective, Behavioral Neuroscience with focus Neurostimulation, Institute of Psychology, Trier University, Trier, Germany; e Institute for Cognitive & Affective Neuroscience, Trier University, Trier, Germany

**Keywords:** MEG, Ischemic stroke, Frontal midline theta, Inhibition, Cognitive control

## Abstract

Frontal midline theta (FMΘ) is a well-established neural marker of cognitive control, generated predominantly in the anterior cingulate cortex (ACC). While reduced theta power has been reported in stroke using EEG, little is known about task-induced FMΘ and its sources in healthy older adults and stroke patients. Understanding FMΘ dynamics is crucial for characterizing post-stroke changes in the neural mechanisms underlying cognitive control and may aid the development of targeted neuromodulation approaches.

In this exploratory study, seventeen healthy older adults and ten chronic ischemic stroke patients performed a Go/NoGo task during magnetoencephalography (MEG). MEG signals were projected into source space using an LCMV beamformer, allowing to localize sources of task-induced FMΘ. At the group level, healthy older adults showed a significant FMΘ cluster localized near the ACC and medial prefrontal cortex. Region-of-interest analyses revealed that healthy older adults exhibited significantly greater task-related FMΘ compared to stroke patients and overall higher baseline theta activity, suggesting impaired upregulation of FMΘ in the patient group during cognitive control.

Overall, the results demonstrate that MEG source localization can capture task-induced FMΘ in healthy older adults at the group level, while modulation in stroke patients appeared more variable and less consistent. Reliable individual localization proved challenging in both groups, suggesting that group-level profiles may currently provide a more feasible basis for stimulation target definition in post-stroke rehabilitation. Future work should focus on validating these findings in larger and more heterogeneous stroke cohorts and on developing methods to bridge the gap between group-level markers and individualized neuromodulation approaches.

## Highlights

• MEG data were recorded from stroke patients and healthy older adults during Go/NoGo.
• Theta sources were localized close to ACC/mPFC.
• Preliminary results indicate reduced frontal midline theta in chronic stroke.
• Effect seems driven by lower theta power at baseline and after NoGo stimuli.
• Challenges in localizing Theta sources in stroke patients are discussed.

## 1 Introduction

Stroke remains one of the leading causes of long-term disability and death worldwide (Grefkes & Fink, 2020). Beyond motor deficits, up to 75% of patients who have suffered from a stroke incident show symptoms of executive dysfunction that often persist into the chronic stroke phase and severely impact activities of daily living. Executive functions such as inhibition, cognitive flexibility, and attentional control are therefore central targets for post-stroke assessment and rehabilitation (Chung et al., 2013).

Frontal midline theta (FMΘ, 4 – 7 Hz) generated predominantly in the anterior cingulate cortex (ACC) has long been investigated as a neural signature of cognitive control across a wide range of task demands (Cavanagh and Frank, 2014, Messel et al., 2021). It has been proposed as a canonical control-related signal that reflects the *need for control* across situations involving conflict, uncertainty, and adaptive adjustment (Cavanagh & Shackman, 2015). Increases in FMΘ are observed in situations that require some aspect of cognitive control, including dual-task training, where higher FMΘ predicted improvements in sustained attention and multitasking (Anguera et al., 2013), inhibitory control tasks such as Go/NoGo and flanker tasks where FMΘ reflects conflict monitoring and successful response suppression (Nayak et al., 2019, Nigbur et al., 2011), and during working memory maintenance where greater FMΘ is linked to more efficient performance (Kardos et al., 2014). This converging evidence underscores FMΘ as a robust mechanistic marker linking neural oscillations to behavioral performance in situations that require flexible allocation of cognitive control. Neurofeedback studies have even shown that upregulation of FMΘ improves performance on cognitive tasks, indicating a close relationship between the two and emphasizing the potential of targeting FMΘ for cognitive rehabilitation (Enriquez-Geppert et al., 2014).

FMΘ power increases are robust in Go/NoGo tasks, making them a central neurophysiological marker for response inhibition, despite debates on whether it reflects inhibition alone or also more general conflict monitoring and attentional allocation (Huster et al., 2013). It is also thought to support the formation and coordination of functional networks that optimize behavior (McLoughlin et al., 2022). This network perspective is particularly relevant in stroke, as lesion effects may not be determined solely by proximity to the anterior cingulate or midcingulate generator, but also by disruption of distributed control architectures—especially interactions between the salience and frontoparietal control networks implicated in executive function (Gratton et al., 2018). Accordingly, when considering lesion location, it may be informative to account not only for distance to the ACC but also for proximity to, or disconnection of, key nodes within these large-scale networks.

While the role of FMΘ in supporting cognitive control is well established in healthy individuals, much less is known about how ischemic stroke can impact FMΘ activity. Evidence from EEG studies suggests that stroke patients show attenuated increases in frontal theta during cognitive tasks compared to healthy adults (Hussain & Park, 2021). Beyond these task-related effects, resting-state studies provide converging evidence for the functional relevance of theta power in stroke. For instance, relative frontal theta power during resting state correlates with Montreal Cognitive Assessment (MoCA) scores in stroke patients, indicating a link between frontal theta and general cognitive performance (Aminov et al., 2017). Additionally, it was found that reduced relative theta power during resting state in acute stage stroke correlated significantly with long-term disability and dependency in activities of daily living at 30 and 90 days post-stroke (Rogers et al., 2020). Finally, stroke patients suffering from spatial attentional deficits showed significantly impaired resting-state frontal theta activity − indicating the importance of long-range integration across the fronto-parietal network for attentional processing (Fellrath et al., 2016).

Together, these findings from EEG studies indicate that theta activity is consistently reduced after stroke and that this reduction contributes to functional impairments. This makes frontal theta a promising target for interventions aiming to restore cognitive function. However, little is known about the source of FMΘ in healthy older adults and chronic stroke populations, essential for targeted cognitive rehabilitation.

In order to investigate the effects of stroke on task-induced FMΘ sources, we employed a Go/NoGo paradigm to probe task-related changes in inhibitory control in both healthy older adults and stroke patients. Using magnetoencephalography (MEG) we mapped sources of FMΘ using a beamformer approach that minimized the influence of tissue conductance. Our aims were threefold: first, to determine whether stroke alters event-related FMΘ modulation during inhibitory control compared to healthy aging; second, to assess whether MEG source localization can reliably identify FMΘ at either the group or individual level, a prerequisite for targeted interventions; and third, to examine whether group differences reflect qualitatively distinct patterns or rather quantitatively reduced modulation, thereby informing whether interventions should be group-based or individualized.

## 2 Methods

### 2.1 Participants

Nineteen healthy older participants and ten chronic ischemic stroke patients were recruited from the local community. One healthy older participant revoked consent to undergo MRI measurement and was therefore excluded. At a later stage, another healthy older participant was removed due to a permanent noise artifact in their MEG data that could not be corrected. This left a final sample of seventeen healthy older adults (M = 62 years, SD = 3 years, self-reported female = 7) and ten stroke patients (M = 67 years, SD = 3 years, self-reported female = 4).

Participants were eligible for the study if they were older than 50 years, had adequate visual, motor, and cognitive abilities to complete all study procedures, provided written informed consent, and reported no history of substance abuse and did not exceed a cutoff score of 22 on the long form of the *Allgemeine Depressionsskala* (ADS-L), a validated German translation of the Center for Epidemiologic Studies Depression Scale (CES-D) (Radloff, 1977, Hautzinger, 1993).

For the patient group, further criteria required a first cerebral or subcortical ischemic infarct in the chronic stage (≥3 months), no history of seizures or unstable medical conditions, and no additional contraindications for magnetic resonance imaging (MRI), such as metal implants or other medical devices. Healthy control participants were required to have no major neurological or psychiatric disorders and no history of psychotropic medication use.

All participants were additionally assessed with the German version of the *Montreal Cognitive Assessment* (MoCA, (Nasreddine et al., 2005)) to characterize cognitive status. Because cognitive impairment is common post-stroke, participants were not excluded if they had low scores on the MoCA. Stroke patients were recruited independent of lesion location or specific behavioral deficits in order to allow for an exploratory approach and to capture a broad spectrum of first-incident chronic ischemic stroke cases.

The study protocol was reviewed and approved by the Medical Research Ethics Board of the University of Oldenburg (approval number: 2021–110). All participants provided written and informed consent prior to participation, and procedures adhered to established ethical standards for research involving human participants, including respect for individual rights, safety, and confidentiality.

### 2.2 MRI measurement and lesion delineation

All data were recorded on site at the Neuroimaging Unit of the Carl von Ossietzky Universität Oldenburg. Data was acquired using a Siemens 3 T Magnetom Prisma Scanner with a 64-channel head coil. For this project, only the T1-weighted (MPRAGE, TR = 2480 ms, echo time TE = 3.3 ms, slice thickness: 0.75 mm) and T2-weighted scans (TR = 5000 ms, TE = 3.9 ms, slice thickness: 1 mm) were used for source localization and lesion delineation. Since this work is embedded in a larger research project, additional DTI and FLAIR scans were also acquired, but not used for the analysis presented in this paper.

Stroke lesion segmentation for all participants was completed using MRIcron v1.0 (Rorden, 2019). Segmentation was completed for the lesion and gliotic tissue separately, then combined into a single, non-overlapping map. This delineation was performed twice by a trained rater to ensure internal consistency, after which all segmentations were reviewed by an experienced neuroradiologist to verify accuracy.

### 2.3 MEG measurement

Data was collected at a sampling rate of 1000 Hz using a 306-channel (102 magnetometers, 204 orthogonal planar gradiometers) Elekta Neuromag Truix system (Elekta Oy, Helsinki, Finland). The system is housed in an electrically and magnetically shielded room (MSR; Vacuumschmelze, Hanau, Germany). All data were collected in an upright, seated position (60° dewar orientation). Prior to the measurement, the location of five head position indicator (HPI) coils and three anatomical landmarks (nasion, left tragus, and right tragus) were digitized along with at least 200 head-shape samples (Fastrak, Polhemus, Colchester, VT, USA).

### 2.4 Experimental task

During MEG recording, participants performed an adapted version of the Go/NoGo task originally developed within the Human Connectome Project (Bookheimer et al., 2019, Lenzini et al., 2019) and previously implemented in our group (Sönmez et al., 2025) with longer inter-stimulus-interval windows (previously an MRI study used windows up to 2400 ms post-stimulus onset) to ensure feasibility for stroke participants and to maximize the number of correct trials for MEG analysis including an appropriate baseline period for event-related analysis. A Go/NoGo task was chosen based on prior work showing that FMΘ is especially pronounced in NoGo trials compared to other interference tasks (Nigbur et al., 2011). This makes the paradigm a well-established elicitor of FMΘ activity. At the same time, the task is cognitively demanding but remains feasible for stroke patients with symptoms of cognitive impairment.

**Fig. 1:**
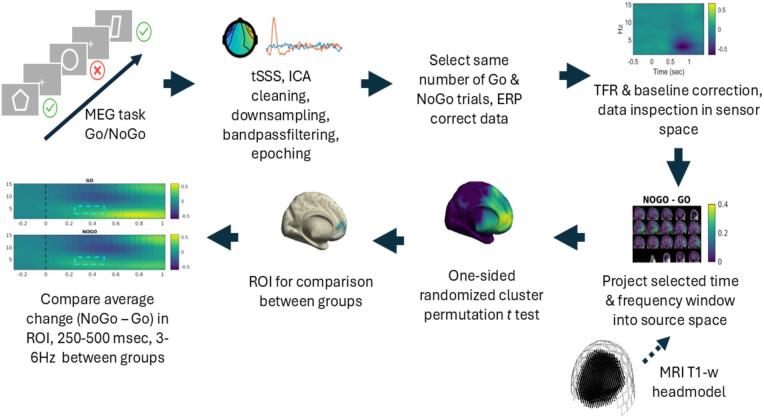
*Overview of the MEG data analysis pipeline for the Go/NoGo task.* Participants completed a Go/NoGo paradigm while MEG data were recorded. Preprocessing included temporal signal space separation (tSSS), ICA-based artifact removal, downsampling, bandpass filtering, and epoching. Equal numbers of correct Go and NoGo trials were subsampled for analysis, and data were ERF corrected (meaning that all trials were averaged and this average was subtracted from each individual trial). Time-frequency representations (TFRs) were computed in sensor space and baseline-corrected. Based on sensor-space analysis and prior literature, a time–frequency window of interest was projected into source space using individual T1-weighted MRI-based head models. Statistical comparisons between trials (NoGo > Go) were conducted using a one-sided cluster-based permutation *t*-test. A follow-up ROI analysis based on the significant cluster identified was conducted to determine if there were significant differences in NoGo and Go trials between groups.

Stimuli consisted of white-outlined geometric shapes on a grey background. Participants were instructed to respond as quickly as possible with a button press of the right-index finger to most shapes (Go trials) and to withhold their response to two specific shapes (NoGo trials). Six shapes (plaques, trapezoids, pentagons, hexagons, octagons, and parallelograms) served as Go stimuli, while squares and circles served as NoGo stimuli (see Fig. 1). Each stimulus was presented on screen for a total of 200 ms after which participants had a time period of 1100 ms to respond. There was a random inter-stimulus interval ranging between 2000–2700 ms to avoid neural adaptation to the stimuli. Participants performed the task in 3 x 10-minute blocks for a total of 504 trials (25% of which were NoGo trials). The task was presented via Presentation software (Version 23.0, Neurobehavioral Systems, Inc., Berkeley, CA, https://www.neurobs.com).

### 2.5 Behavioral analyses

To investigate group differences in performance (accuracy on Go and NoGo trials, response times, and d′), we compared healthy controls and stroke patients using one-tailed Mann–Whitney U rank-sum tests. One-tailed testing was chosen because we had an a priori directional hypothesis that stroke patients would show poorer inhibitory control, reflected in lower accuracy, longer response times, and reduced sensitivity compared to healthy older adults. The use of a non-parametric independent-samples test was motivated by the small sample size and the potential for our data being skewed due to outliers.

We calculated d′ as a sensitivity index to measure participants’ ability to discriminate Go from NoGo stimuli (Hautus, 1995, Hautus et al., 2021). Unlike raw accuracy, d′ accounts for both hits (correct responses to target trials) and false alarms (incorrect responses to non-target trials) providing a more robust estimate of inhibitory control. Hits and false alarms were first expressed as proportions, with extreme values corrected using the log-linear rule, to avoid infinite z-scores for extreme hit or false-alarm rates and to reduce small-sample bias:

$$\hat{H}=\frac{H+0.5}{N_{signal}+1}$$

$$\hat{F}=\frac{F+0.5}{N_{noise}+1}$$

where H and F denote the number of hits and false alarms, and $N_{signal}$ and $N_{noise}$ are the total number of signal and noise trials, respectively. Sensitivity was then calculated as

$$d^{\prime }=\Phi^{-1}\left(\hat{H}\right)-\Phi^{-1}\left(\hat{F}\right)$$

with $\Phi^{-1}$ representing the inverse cumulative distribution function of the standard normal distribution. Higher values of *d′* indicate greater ability to discriminate between signal and noise trials. Values from both groups were also subjected to a one-tailed Mann-Whitney U rank sum test.

To further assess the robustness of the observed behavioral group differences, we applied a non-parametric bootstrap procedure (10,000 resamples with replacement within each group). For each resample, we recomputed the Mann-Whitney U statistic, the associated rank-biserial correlation, and the corresponding p-value. Percentile-based 95% confidence intervals, the proportion of resamples preserving the original effect direction, and the proportion yielding p < 0.05 (“p-stability”) were used to quantify the stability of the behavioral findings. We also assessed the robustness of our results using a leave-one-out analysis, in which the full analysis pipeline was repeated while systematically excluding one participant at a time to evaluate the influence of individual subjects on the observed effects.

To assess the relationship between behavioral performance and neural measures, correlation analyses were performed between d′ and (a) cluster size and (b) change in frontal midline theta, Δ FMΘ. Because several variables deviated from normality, associations were quantified using Spearman’s rank correlation coefficient. All tests were two-tailed, and statistical significance was evaluated at α = 0.05. Figures can be found in Supplementary Fig. S1.

### 2.6 MEG analysis

#### 2.6.1 Pre-Processing

To suppress external noise, a spatiotemporal signal space separation method (tSSS) was applied with parameters for harmonic extension order set to L_in_ = 8 and L_out_ = 3, and a correlation cutoff of 0.98, using the MaxFilter implementation in MNE-Python v1.10.0 (Gramfort, 2013). This technique separates signal components originating inside the MEG helmet from those outside, while simultaneously correcting for head movements using continuous head position indicator (cHPI) signals (Nenonen et al., 2012, Taulu et al., 2005, Taulu and Simola, 2006). Each participants head position was transformed to the initial head position in the first block of the experiment (Larson et al., 2025).

As an additional quality assurance measure, translational and rotational head motion indices were derived from cHPI signals and compared between groups as part of data quality control. No significant group differences were observed for either positional or rotational motion (rotation p = 0.2147, translation p = 0.21932), suggesting comparable head stability across groups.

Subsequent analysis was performed with Matlab R2023a (The MathWorks, Inc. Natick, MA, USA) and Fieldtrip-20250402 (Oostenveld et al., 2011). Data were downsampled to 250 Hz and segmented into 2 s windows in preparation for ICA. Segments with high variance (> 4 SD) were excluded. ICA components corresponding to eye blinks, muscle movement, and cardiac components were identified as artifacts and removed before back projecting the data into sensor space. An average of 3.2 components (SD = 1.2) were rejected for healthy controls, and an average of 3.6 components (SD = 1.43) for stroke patients – both groups had a maximum of 6 components and a minimum of 2 components removed.

#### 2.6.2 Sensor-Space analysis

Once the raw data were processed with ICA, only magnetometer data were retained for further analysis. Data were resampled to 500 Hz and butterworth IIR bandpass filtered (1 – 30 Hz, filter order 4, zero-phase forward and reverse) prior to being epoched into 3 s segments (−1.5 s to + 1.5 s around stimulus onset). Epochs were rejected if the participant’s response was incorrect, if it was too early (anticipatory reactions ≤ 100 ms post stimulus onset), or if the variance in the average signal of the epoch exceeded 2 SD of the mean variance for all epochs (removing epochs with abnormally high noise). We applied a lower sample rate for the ICA cleaning to improve computation time and had more liberal definitions of SD cutoff to retain some artifacts for component definitions. For time–frequency analysis, epochs were zero-padded to 8 s to improve spectral resolution.

We retained an average of 354.89 Go trials for our control group (SD = 23.54) and 351.7 Go trials for our stroke group (SD = 21.63). The number of Go trials were randomly subsampled to match the number of NoGo trials to prevent bias in our beamformer analysis and allow for fair comparison between both trial types. Because of this, we retained a final average of 110.39 trials for our control group (SD = 10.36) and 108.2 trials for our stroke group (SD = 13.66) for both Go and NoGo conditions. Each trial was then ERF corrected, wherein the average ERF was subtracted. This was performed separately for the Go and NoGo trials to isolate induced activity (Beatty et al., 2020, Cohen and Donner, 2013, Herrmann et al., 2014). Induced activity allows us to look at time-locked synchronization of theta oscillations without the necessity of the data to be phase-locked across trials, and tends to correlate more with trial-to-trial variances in reaction time and behavior (Cohen & Donner, 2013).

Since FMΘ activity has been shown on the group level for older healthy participants in prior EEG studies, we began by inspecting grand averaged data (Cabeza et al., 2018, Cummins and Finnigan, 2007, Kardos et al., 2014). A multi-taper time–frequency convolution was calculated on each trial using a Hanning taper with a spectral power output for frequencies between 1 to 15 Hz, in 0.5 Hz steps and –1.5 to + 1.5 s, in 50 ms steps. Then, the output was averaged for each trial and corrected relative to change with a selected baseline of −1500 to −500 ms around stimulus onset.

A sliding 100 ms window between + 200 ms and + 600 ms and a sliding 1 Hz frequency band window between 3 Hz and 8 Hz was used for data inspection. These windows were selected based on prior works indicating a reasonable range for slowing of theta oscillations and delays in activity due to age (Van De Vijver et al., 2014).

To complement the cluster-based analyses, theta-band power during baseline and active periods was summarized using boxplots, and corresponding frequency spectra were extracted from pre-existing source space TFR data. To further contextualize inter-individual variability, event-related fields (ERFs) and individual power spectra plots are presented in Supplementary Fig. S2.

All Go data were grand averaged and then subtracted from the grand averaged NoGo data. Topographies for each window (5 1-Hz frequency bands x 4 100-ms time windows) were visualized to identify a time range and frequency range where the largest difference between the two trials could be observed. Based on these topographies, we identified a time–frequency window where theta power was higher during NoGo than Go trials. The strongest group-level effect was observed between 3–6 Hz and 250–500 ms post-stimulus. This window was therefore selected for subsequent analyses. Because individual participants showed substantial variability in the exact timing and frequency of theta activity, it was not possible to define subject-specific windows, so all analyses were conducted using this group-defined window. This also meant that FMΘ could not be found reliably on the individual level.

#### 2.6.3 Source-Space analysis

A linearly constrained minimum variance (LCMV) beamformer for all magnetometers with a regularization λ of 5% was used to project ERF corrected, epoched data into source space. A common spatial filter was computed from the covariance matrix estimated across all segments of Go and NoGo trials. Individual T1-weighted MRIs were used to compute single-shell head models for each participant that were then co-registered to match positioning within the MEG helmet. To standardize source location, a regular 6 mm 3D grid warped into the Montreal Neurological Institute (MNI) template was used as source-space for forward modeling. Spatial filters were then used to compute virtual time series of each trial at each source location.

To validate our time window of interest, time–frequency differences between Go and NoGo conditions were assessed using a nonparametric cluster-based permutation test implemented in FieldTrip. For each subject, source-level time–frequency representations were first restricted to the frequency range of 1–10 Hz and the time window of 0–1 s post-stimulus. Condition differences were evaluated using paired-sample t-statistics at each source location, frequency, and time point. To control for multiple comparisons across the three-dimensional space–time–frequency domain, adjacent samples exceeding a cluster-forming threshold (α = 0.05, one-tailed) were grouped into clusters, and cluster-level statistics were computed as the sum of t-values within each cluster. Statistical significance was determined using a Monte Carlo permutation procedure (5000 permutations), in which condition labels were randomly exchanged within subjects to generate a null distribution of maximal cluster statistics. Observed clusters were considered significant if their cluster-level probability was below p < 0.05.

No significant clusters were found in either group. Nevertheless, inspection of the t-value maps revealed that healthy controls exhibited the strongest positive NoGo–Go differences in the theta range approximately 200–400 ms post-stimulus onset, and between 3–6 Hz, whereas no comparable pattern was observed in the stroke group. This validates our initial time/frequency window selection and we have included it as validation of our selection. Results can be seen in supplementary Fig. S2.

To localize sources of task-induced theta activity, source time courses were submitted to a TFR analyses matching the one described earlier in sensor space. Spectral estimates were computed with a Hanning taper in the frequency range from 1 to 15 Hz in steps of 0.5 Hz. All data was corrected relative to a baseline window of −1500 to −500 ms around stimulus onset, calculated as relative change (fractional change relative to baseline). This baseline period was selected due to the sliding window applied for spectral estimates to avoid inclusion of post-stimulus activity to the pre-stimulus power estimates. A fixed time window of 1 s was applied for all frequencies. The data were zero-padded to 8 s to improve spectral resolution. TFRs were calculated across the time interval from –1.5 s to + 1.5 s around the event onset, with sample windows of 50 ms. The analysis was conducted separately for Go and NoGo trials, and relative power estimates for each individual were retained for subsequent statistical analysis. The averaged relative power from the selected frequency (3–––6 Hz) and time window (250 – 500 ms post-stimulus) was used as input for statistical comparison.

To test if there was a significant increase in theta activity during the NoGo trials compared to the Go trials without predefining the expected location of effects in advance and while accounting for multiple comparisons, a one-sided dependent samples permutation cluster *t* test with Monte Carlo estimates was performed with 10,000 random permutations to estimate *p* values (Maris & Oostenveld, 2007). For each permutation, the maximum cluster-level test statistic (i.e., the sum of t-values within each spatially contiguous cluster, “cluster mass”) was stored to form the null distribution against which the observed cluster statistic was compared. Clusters exceeding the 95th percentile of this null distribution (p < 0.05) were used as regions of interest (ROI) in the follow-up TFR analysis of group differences.

### 2.7 ROI analysis

Once an ROI was identified in source space for our healthy older cohort, indexes corresponding to the ROI in individual source space were extracted from the pre-existing non-baseline corrected TFRs. Mean power values were computed by averaging across all virtual channels within the ROI.

For each participant, theta power (3–6 Hz) was extracted separately for Go and NoGo trials and segmented into baseline (−1500 to −500 ms) and active (250 to 500 ms) task periods. Within each time window, power values were averaged across frequencies within the theta band and across all time points in the selected interval, yielding a single summary value per subject, condition, and state.

Theta power values were submitted to a linear mixed-effects model with within-subject factors State (baseline, active) and Condition (Go, NoGo), and the between-subject factor Group (control, stroke). The model was fitted in MATLAB R2023a (The MathWorks, Inc. Natick, MA, USA) using the fitlme function to estimate fixed effects of Group, State, Condition, and their interactions, with a random intercept for Subject to account for repeated measures (Power ∼ Group × State × Condition + (1|Subject)). Type III F-tests for fixed effects were obtained using anova(lme), and partial eta squared (ηp^2^) was calculated from the corresponding F-statistics and degrees of freedom to quantify effect sizes.

To further characterize significant effects, nonparametric follow-up tests were performed. Within-group differences between baseline and active periods were assessed using paired Wilcoxon signed-rank tests. Between-group differences at each task state were evaluated using Wilcoxon rank-sum tests.

Additionally, we averaged the baseline-corrected relative change in power in the corresponding voxels for all participants from the time (250 ms – 500 ms) and frequency (3 – 6 Hz) window of interest and extracted a single value for the NoGo and Go trials separately. This yielded two trial-specific values for each participant, one representing event-related activity during Go trials, and one representing event-related activity during NoGo trials – now corresponding to our ROI. To quantify modulation related to inhibitory control, we computed the difference between NoGo and Go values, treating this as a measure of theta change associated with inhibition (Δ FMΘ).

Group differences in Δ FMΘ between healthy and stroke participants were then assessed using complementary statistical approaches. First, to test for differences in central tendency without assuming normality due to our smaller sample size, we performed a Mann–Whitney U rank-sum test. Then, a Kolmogorov–Smirnov (KS) test was conducted to evaluate potential differences in the overall distributional shape between groups.

To assess the stability of the observed group difference in Δ FMΘ, we performed a non-parametric bootstrap (10,000 resamples with replacement within each group). For each resample, we recomputed the median group difference (Controls − Stroke), the rank-biserial correlation associated with the Mann-Whitney *U* test, and the corresponding p-value. We report percentile 95% confidence intervals, the proportion of resamples preserving the effect direction, and the proportion yielding p < 0.05 (“p-stability”). Here, leave-one-out analysis was again used to estimate the influence of individual subjects on the observed effects.

TFR plots corresponding to our ROI and stroke lesion maps (including volume of lesion) are included in supplementary Fig. S4.

## 3 Results

### 3.1 Participant Characteristics

Groups were comparable in age, sex distribution, and years of education. Healthy older participants (M = 11.29 years, SD = 1.16) and stroke patients (M = 12 years, SD = 1.22, p = 0.12) had years of education ranging between 10 and 13 years for both groups. Stroke patients were additionally required to have experienced an ischemic stroke at least three months prior to participation, ensuring that all participants were in the chronic phase.

Depression scores (ADS-L) showed no significant differences between groups (HC: Median (Mdn) = 5, IQR = 5; stroke: Mdn = 9.5, IQR = 12; *U* = 240.5, *z* = -0.18, rank-biserial correlation *r* = -0.23, *p* = 0.17). In contrast, MoCA scores were significantly higher in healthy adults (Mdn = 28.5, IQR = 2) compared to stroke patients (Mdn = 25.5, IQR = 3; *U* = 316, *z* = 0.50, rank-biserial correlation *r* = 0.61, *p* = 0.004).

**Fig. 2:**
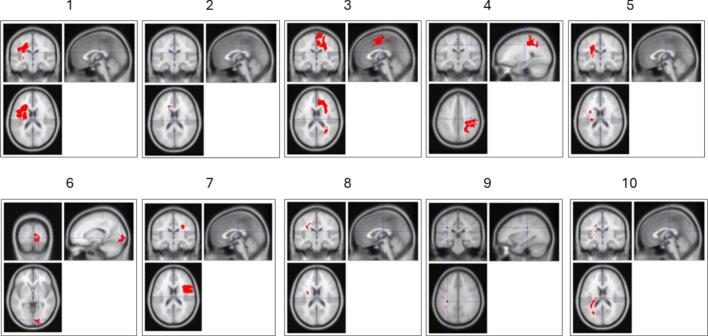
*Overview of Stroke Lesion Maps across patients.* Lesion maps include gliotic tissue and lesion tissue and were warped to the MNI avg152T1 template provided by SPM12 in coronal, sagittal and axial views to ensure patient anonymity.

Fig. 2 visualizes the individual lesion masks for each patient. Lesions were most commonly located in frontal or parietal regions but showed considerable heterogeneity in both location and size. All patients were in the chronic stage, with stroke incidence (M = 7.3 years, SD = 4.97) dating back between 2 to 16 years prior to participation.

### 3.2 Behavioral results

**Fig. 3:**
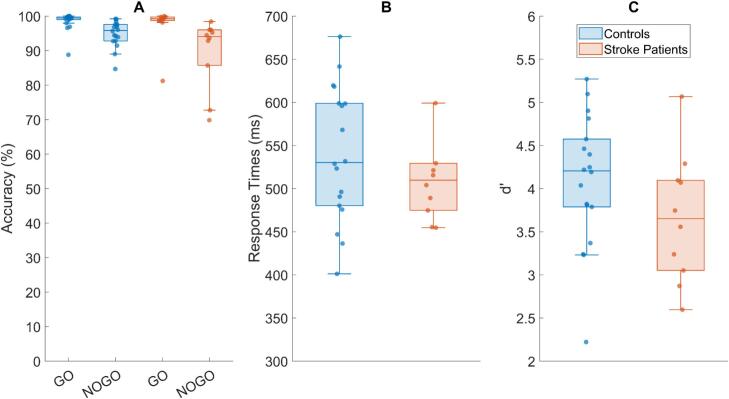
*Performance in the Go/NoGo task.* (A) Accuracy in Go and NoGo trials for healthy participants (blue) and stroke patients (red). Both groups performed with high accuracy, although stroke patients showed numerically lower accuracy in NoGo trials (B) Response times in Go trials for both groups with no significant difference. (c) Sensitivity (d’) in the Go/NoGo task showing numerically lower sensitivity in stroke patients. Data distributions are displayed as boxplots, where the central line represents the median, the box edges indicate the first and third quartiles (interquartile range, IQR), whiskers extend to the most extreme data points within 1.5 × IQR, and values outside this range are plotted individually as outliers. Dots represent data of individual participants/patients. (For interpretation of the references to colour in this figure legend, the reader is referred to the web version of this article.)

On Go trials, healthy controls responded with high accuracy (*Mdn* = 99.44%, *IQR =* 0.78), which did not differ significantly from the performance of stroke patients (*Mdn* = 99.21%, *IQR* = 1.06, *U* = 108, *z* = 0.818, rank-biserial correlation *r* = 0.194, *p* = 0.21). Accuracy was also high in both groups on NoGo trials (controls: Mdn = 95.84%, IQR = 4.84; stroke patients: Mdn = 94.05%, IQR = 10.32). Stroke patients nevertheless showed numerically lower accuracy and greater variability, with the accuracy difference trending towards poorer NoGo performance (U = 120, z = 1.39, rank-biserial correlation r = 0.33, p = 0.08; Fig. 3a).

Bootstrap analyses of behavioral accuracy indicated a group effect (r = 0.19, p = 0.41) with a notable directional consistency (Makowski et al., 2019) of 82.1% across resamples. Although the wide 95% confidence interval (–0.25 to 0.62) reflects the inherent variability of a clinical sample, the high frequency of resamples following the same direction suggests a stable underlying trend. While leave-one-out analysis showed that the group difference did not reach significance in any iteration, bootstrapping results reached significance 12.7% of the time.

For NoGo accuracy, bootstrap analyses revealed a small-to-moderate group effect (r = 0.33, p = 0.16). Despite a wide 95% confidence interval (−0.13 to 0.71), the effect demonstrated strong directional consistency, with 84.7% of resamples showing the same direction of group difference. This high level of consistency indicates a stable underlying trend across resampled datasets. Statistical significance was observed in 28.8% of resamples, reflecting variability associated with limited sample size rather than inconsistent effects. Leave-one-out analyses did not yield significant results, further highlighting constraints in statistical power, warranting further investigation in larger cohorts.

Median response times on correct Go trials were 530.21 ms (*IQR* = 118.71) for controls and 509.80 ms (*IQR* = 54.66) for stroke patients with no significant group difference (*U* = 104, *z* = 0.695, rank-biserial correlation *r* = 0.16, *p* = 0.76; Fig. 3b).

For response times, the observed group difference was small (r = 0.16, p = 0.52). Although the 95% confidence interval was wide (−0.29 to 0.60), bootstrap analyses revealed consistent directional patterns, with 82.2% of resamples showing the same direction of group difference. This moderate-to-high directional consistency suggests a stable trend across resampled datasets. Statistical significance was observed in 10.1% of resamples, likely reflecting limited statistical power rather than inconsistent effects. Leave-one-out analyses did not yield robust effects, further highlighting sample-size constraints.

Sensitivity (d’) was numerically lower in stroke patients (*Mdn* = 3.65, *IQR* = 1.04) compared to healthy older adults (*Mdn* = 4.21, *IQR* = 0.79), a tendency that approached but did not reach significance (*U* = 124, *z* = 1.61, rank-biserial correlation *r* = 0.38, *p* = 0.054; Fig. 3c).

For sensitivity (d′), the observed group effect was moderate (r = 0.38, p = 0.11), with higher values in controls. Although the 95% confidence interval was wide (−0.07 to 0.77), bootstrap analyses revealed very high directional consistency, with 92.7% of resamples showing the same group difference. This strong consistency suggests a stable underlying effect across resampled datasets. Statistical significance was observed in 36% of resamples, reflecting variability associated with limited sample size. Leave-one-out analyses reached significance in 1 of 10 iterations, further highlighting power constraints.

Correlation analyses revealed no significant associations between changes in frontal midline oscillatory activity (Δ FMΘ) and behavioral sensitivity (d′) across all participants (r = 0.09, p = 0.647), in the control group (r =  − 0.19, p = 0.446), or in the stroke group (r = 0.25, p = 0.492). Similarly, lesion size was not significantly correlated with d′ in stroke patients (r = 0.27, p = 0.448). Although small-to-moderate positive trends were observed in the stroke group and in relation to lesion size, none of these relationships reached statistical significance, indicating limited evidence for a direct association between neural and behavioral measures in the present sample (Supplementary Fig. S1).

### 3.3 Group-Level FMΘ

To address our first question, whether stroke alters event-related FMΘ modulation during inhibitory control we first compared differences in theta power between NoGo and Go trials within each group. To this end, we computed whole-brain t-maps with dependent samples cluster permutation t tests comparing activity between 3–––6 Hz, 250 – 500 ms.

**Fig. 4:**
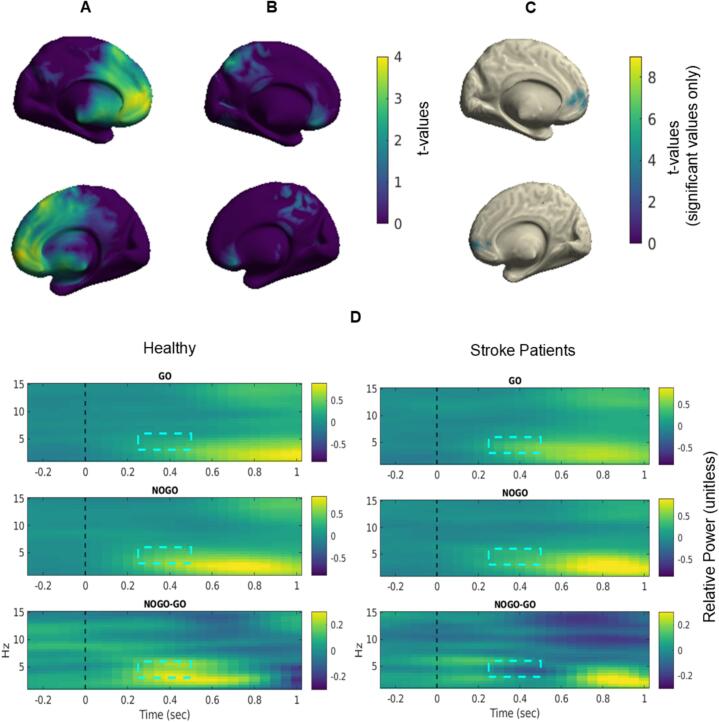
*Source of task-induced Theta.* Un-thresholded T-value map obtained from an independent samples cluster permutation *t*-test comparing the difference in task-induced theta activity between the NoGo and Go conditions in (A) Healthy older adults, and (B) Stroke patients. While healthy older adults showed significant increases in theta located near the ACC/medial prefrontal cortex, stroke patients did not show this same pattern. (C) Thresholded T-value map depicting the significant cluster showing stronger theta power in the NoGo relative to the Go trials identified in healthy older adults. (D) Source-level TFRs extracted from the cluster shown in A for healthy older adults and stroke patients for Go, NoGo, and the NoGo-Go contrast with the analysis window indicated by a box. The time–frequency window was selected based on a combination of prior work by Van De Vijver et. al. (2014) showing a slowing of task-related theta during aging and our analysis of event-related changes in theta in signal space.

Only one significant cluster was found for healthy controls (p_cluster_ = 0.0212) with a peak t-value of 4.04 (Cohen’s d = 0.98) located at MNI coordinates [-1.5 5.0 1.0], near the ACC and medial prefrontal cortex. Stroke patients showed no significant clusters (Fig. 4a–c). Within this ROI we can observe a pronounced NoGo–Go difference in healthy controls and a reduced but comparable pattern in stroke patients (see Fig. 4d for an overview of task induced spectral changes within the ROI).

### 3.4 ROI analysis

Since a significant difference between conditions was found for healthy older participants, we wanted to explore if there were group differences in this specific region. To explore theta changes associated with the ROI found in healthy older participants, we compared the change in FMΘ between the groups only from this region.

For the first ROI analysis, initial nonparametric comparisons indicated that stroke patients exhibited lower theta power compared to controls already during baseline periods and showed reduced theta power during NoGo trials. To formally evaluate these effects within a unified factorial framework, a three-factor linear mixed-effects model was conducted with Group (control, stroke) as a between-subject factor and State (baseline, active) and Condition (Go, NoGo) as within-subject factors.

The linear mixed-effects model did not yield a significant 3-way interaction (F(1,100) = 2.82, p = 0.096, ηp^2^ = 0.027, *small effect size*) (Lakens, 2013), indicating that the combined modulation of theta power by State and Condition did not differ reliably between groups.

We therefore proceeded to examine the two-way interactions. A significant Group × Condition interaction F(1,100) = 3.97, p = 0.049, ηp^2^ = 0.038, *small effect size*) was found, indicating that group differences depended on task condition. Additionally, a State × Condition interaction (F(1,100) = 7.36, p = 0.008, ηp^2^ = 0.069, *medium effect size*) was observed, reflecting differential baseline-to-active changes across task conditions. The Group × State interaction was not significant (F(1,100) = 0.04, p = 0.835, ηp^2^ < 0.001, *negligible effect size*).

Given the significant two-way interactions, follow-up comparisons were conducted to characterize these effects. Within-group comparisons confirmed robust baseline-to-active increases in both groups. For Go trials, significant increases were observed in controls (p = 0.0018, rank-biserial correlation r = 0.84) and stroke patients (p = 0.0039, rank-biserial correlation r = 0.96). Similarly, for NoGo trials, significant increases were present in controls (p = 0.0002, rank-biserial correlation r = 0.99) and stroke patients (p = 0.0020, rank-biserial correlation r = 1.00), all reflecting large effects.

**Fig. 5:**
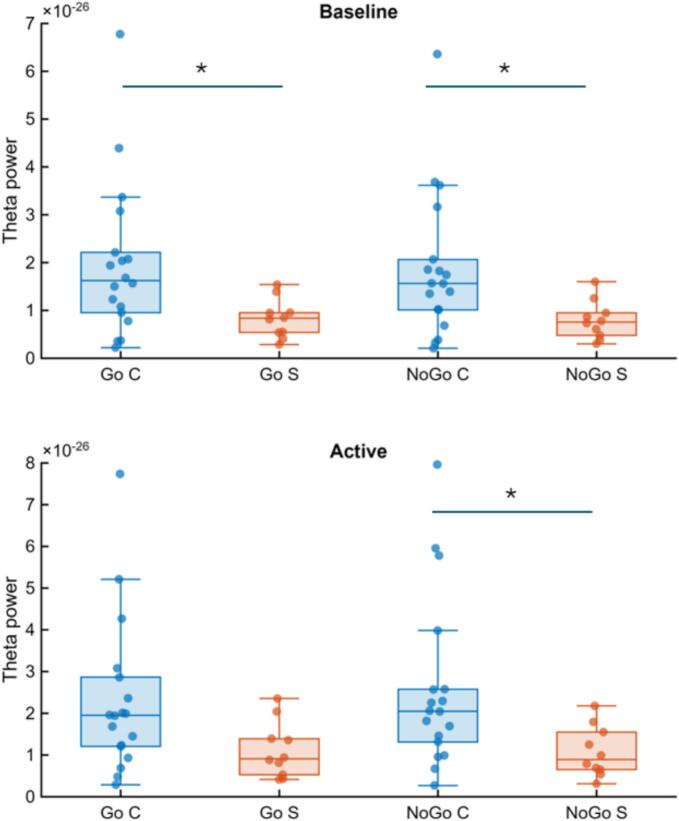
*Group differences in Go and NoGo Baseline and Active Theta.* Boxes indicate median and interquartile range, and dots represent individual participants. Both groups showed significant baseline-to-active increases, whereas stroke patients exhibited reduced theta power relative to controls, particularly during NoGo active trials. *p < 0.05. All results from non-parametric tests showed significant results with the exception of Go active period between groups (0.058).

Between-group comparisons clarified the significant Group × Condition interaction. At baseline, stroke patients exhibited lower theta power than controls in both Go (p = 0.020, rank-biserial correlation r = 0.44, *medium effect size*) and NoGo conditions (p = 0.020, rank-biserial correlation r = 0.44, *medium effect size*), indicating moderate baseline group differences. During task engagement, group differences were condition-dependent: stroke patients exhibited significantly lower active theta power compared to controls during NoGo trials (p = 0.014, rank-biserial correlation r = 0.47, *medium effect size*), whereas the corresponding Go active difference did not reach statistically significance (p = 0.058, rank-biserial correlation r = 0.36, *medium effect size)* (Fig. 5).

Finally, significant main effects of Group (F(1,100) = 4.38, p = 0.039, ηp^2^ = 0.042), State (F(1,100) = 10.08, p = 0.002, ηp^2^ = 0.092), and Condition (F(1,100) = 8.22, p = 0.005, ηp^2^ = 0.076) were observed. However, given the presence of significant two-way interactions, these main effects should be interpreted in the context of the interaction patterns described above.

**Fig. 6:**
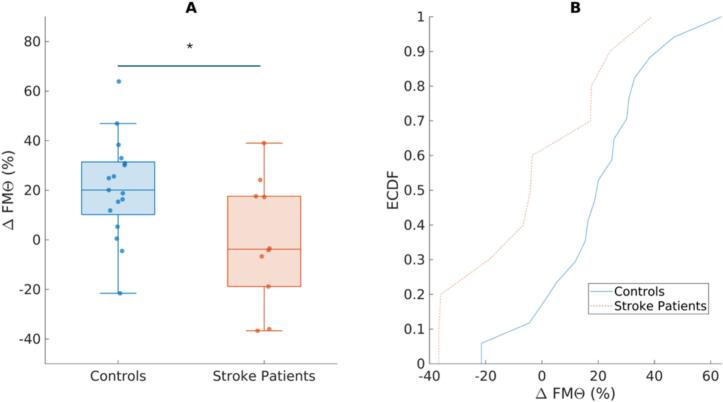
*Group differences in Δ FMΘ activity ROI*. (A) Boxplots indicate the distribution of induced theta activity in the ROI across participants for healthy controls and stroke patients. Healthy controls exhibit significantly larger task-induced theta activity compared to stroke patients (*p* = 0.02). The central line represents the median, the box edges indicate the IQR and whiskers extend to the most extreme data points within 1.5 × IQR, and values outside this range are plotted individually as outliers. (B) describes the distributions of data between the two groups. Here, there were no significant differences in the empirical cumulative distribution functions (ECDF) of either group − which serves as a measure of the proportion of observations less than or equal to each value as a cumulative probablity between 0 and 1. This indicates that despite the difference in group sizes, the difference we observe is due to a greater over all shift in Δ FMΘ, and not just a distributional difference in data points between the two groups.

For our second analysis, compared to stroke patients (*Mdn* = -0.04, *IQR* = 0.36), healthy controls (*Mdn* = 0.20, *IQR* = 0.21) exhibited significantly greater Δ FMΘ in the 250–500 ms interval (NoGo–Go) in the ROI (Fig. 6a). A non-parametric Mann-Whitney U rank-sum test (*U* = 126, *z* = 2.03, *p* = 0.021, rank-biserial correlation *r* = 0.48) corroborated these findings (Fig. 6a).

Additionally, a Kolmogorov–Smirnov test, which measures the largest difference between empirical cumulative distribution functions (ECDF), a measure of the proportion of observations less than or equal to each value as a cumulative probability between 0 and 1, did not indicate a significant difference in the overall distributional shapes between groups (*D* = 0.48, *p* = 0.072; Fig. 6b). The only trend observed was that the distribution of Δ FMΘ values for stroke patients tended to be lower in overall value compared to healthy controls but both groups tended to have their values concentrated in similar ranges.

Bootstrap analysis of the Δ FMΘ group effect demonstrated a high directional consistency, with the effect (Controls > Stroke) being preserved in 94% of all resamples. Although the high stringency of the bootstrap led to significant p-values in only 53.6% of iterations, the robustness of the effect direction supports the hypothesis of reduced theta modulation in stroke patients. The leave-one-out analysis reached significance in the majority of iterations (6 out of 10), although the absolute p-value is sensitive to the exact composition of the clinical group, and warrants further investigation with a larger cohort.

## 4 Discussion

We investigated task-induced FMΘ activity during inhibitory control in healthy older adults and stroke patients using a Go/NoGo paradigm and MEG source localization. Three main findings emerged. First, only healthy older adults showed significant increases in FMΘ during NoGo compared to Go trials, whereas stroke patients did not exhibit comparable modulation. Second, due to the beamforming advantages in MEG data, we were able to localize FMΘ sources at the group level for healthy older adults, with peak activity near the ACC and medial prefrontal cortex. Third, group differences were best explained by a shift in central tendency rather than qualitatively distinct patterns, suggesting that stroke patients retain a similar but attenuated FMΘ profile relative to healthy controls.

Our sample of stroke patients showed reduced cognitive performance relative to healthy older adults, as reflected in significantly lower MoCA scores. This finding is consistent with prior work reporting high rates of cognitive impairment in chronic stroke patients (Aminov et al., 2017). In the Go/NoGo task, both groups performed with high accuracy.

Nevertheless, stroke patients exhibited numerically lower accuracy in NoGo trials and lower sensitivity (d′), although neither reached statistical significance. This pattern is to some extent consistent with larger behavioral studies reporting that a substantial proportion of stroke patients, particularly those with right-hemisphere lesions, show prolonged response times and increased false alarms in Go/NoGo tasks, indicating impairments in inhibitory control (Spaccavento et al., 2019).

At the group level, healthy controls showed a NoGo-related theta increase localized to the anterior cingulate and medial prefrontal cortex. Prior MEG studies of frontal midline theta in young adults performing mental calculation or working memory tasks reported midfrontal sources that were, however, more dorsal (Ishii et al., 1999, Ishii et al., 2014, Rhodes et al., 2023). The slightly more ventral localization in our data may therefore reflect task-specific demands of inhibitory control or age-related shifts in the spatial profile of FMΘ. Recent work has shown that what is typically identified as FMΘ reflects the contribution of multiple partially independent generators within medial prefrontal and cingulate networks, each associated with different aspects of cognitive control (Zuure et al., 2020). This offers a plausible explanation for why the precise localization of FMΘ may vary across tasks. Supporting this interpretation, we previously observed NoGo-related activity in a ventral ACC/mPFC region during fMRI under vagus nerve stimulation using the same Go/NoGo paradigm, suggesting that this region may play a specific role in inhibitory control (Sönmez et al., 2025). A further, though more speculative, possibility is that aging could alter the relative contributions of FMΘ generators, leading to subtle spatial shifts in apparent source localization. However, direct evidence for such age-related changes is currently limited.

In contrast to healthy older adults, stroke patients in our study showed a marked reduction of task-related FMΘ. During both baseline and active periods, theta power was significantly reduced in our stroke cohort. All iterations reached statistical significance with moderate to large effect sizes, except for the group differences in frontal theta activity during the Go active period. This indicates a disruption in theta that exists already at the baseline and that plateaus even during a less challenging task (Go condition). Previous work has shown that resting-state oscillatory activity is clinically relevant after stroke, with power in the alpha, beta, and gamma bands predicting cognitive outcomes (Snyder et al., 2021), and resting-state theta power correlating with performance on the MoCA (Schleiger et al., 2017). This aligns with our results, with the new addition of impairment during active task performance.

Underlying mechanisms for these disruptions have been hypothesized to originate from ischemic disruptions causing shifts towards excess delta activity that subsequently reduces or attenuates FMΘ (Aminov et al., 2017). Additionally, larger network disruptions caused by lesions can blunt theta increases during tasks leading to weaker reactivity and reduced processing capacity (Fellrath et al., 2016, Hussain and Park, 2021).

Previous lesion work indicates that damage to medial frontal regions such as the ACC can directly impair reward-guided decision-making and inhibitory control (Swick and Turken, 2002, Vázquez et al., 2024). Recent animal studies similarly demonstrate that ACC disruptions reduce theta activity, impair decision-making, and increase impulsivity (Ye et al., 2023). It should be noted, however, that our patient group did not share a uniform lesion site (Fig. 2); lesions were heterogeneous in location and extent. The consistent reduction of FMΘ across this heterogeneous sample may suggest that FMΘ does not rely on a single cortical generator, but rather reflects distributed network dynamics that can be disrupted by lesions at different nodes (Zuure et al., 2020). However, to date, there has been no study directly linking lesion location and impairment of FMΘ activity in stroke patients. Hence, while were able to show that stroke alters event-related FMΘ modulation during inhibitory control and that MEG source localization can reliably identify FMΘ at group level in healthy old adults, our attempts to localize FMΘ at the individual level did not yield consistent midfrontal sources, and group-level localization was not feasible in the stroke patient group. This limits the level of individual tailoring available for customized rehabilitation options for both patients and healthy adults.

For example, non-invasive brain stimulation techniques such as transcranial direct (tDCS) or alternating current stimulation (tACS) are increasingly tested for the treatment of neurological and psychiatric conditions (Elyamany et al., 2021, Pipatsrisawat et al., 2022, Savelon et al., 2024) and show potential for rehabilitation (Fedorov et al., 2010, Maggio et al., 2024, Shen et al., 2022). Given the reductions in FMΘ in our stroke patient group, tACS might be a particularly interesting intervention approach to restore reduced FMΘ in stroke patients due to its ability to increase the power of brain oscillations for extended periods of time (Kasten et al., 2016, Kasten and Herrmann, 2017, Neuling et al., 2013), by inducing neuroplastic changes (Vossen et al., 2015, Wischnewski et al., 2019, Zaehle et al., 2010).

More recent work suggests that optimizing stimulation protocols can enhance the effectiveness of interventions (Ovadia-Caro et al., 2019, van der Cruijsen et al., 2022), with individualized approaches incorporating source localization and e-field modeling showing particular promise for improving outcomes (Kasten et al., 2019). Specifically, tACs has been used to target FMΘ in healthy populations to modulate working memory (Chander et al., 2016, Jaušovec and Jaušovec, 2014), error and conflict processing (Fusco et al., 2018), and inhibition (Klírová et al., 2021).

Several methodological factors should be considered when interpreting the present findings. First, increases in low-frequency power during task performance can, in principle, reflect the spectral representation of ERFs as slow evoked components inherently contribute power in the delta–theta range. In the present study, however, phase-locked activity was removed prior to time–frequency analysis, such that the reported effects reflect induced (non–phase-locked) activity rather than purely evoked responses (Herrmann et al., 2014). While this substantially reduces the likelihood that the findings are driven by ERF contamination, we cannot entirely exclude that residual low-frequency components may contribute to the observed 3–6 Hz modulation. Nevertheless, the persistence of the effect after ERF correction supports the interpretation of altered oscillatory dynamics rather than a simple reflection of evoked potentials.

Second, our analyses focused on a predefined 3–6 Hz frequency range to maintain consistency with prior works showing significant frequency slowing in the theta range in older individuals (Van De Vijver et al., 2014). We did not formally test whether the observed effects were strictly confined to this range or extended into adjacent frequency bands in this specific study. It therefore remains possible that the modulation reflects broader low-frequency changes rather than a narrowly defined theta-band effect. Future studies using broader, data-driven spectral comparisons could clarify the frequency specificity of task-related modulation in both healthy older adults and stroke patients.

Additionally, behavioral performance in both groups approached ceiling levels, indicating that the Go/NoGo task may not have been sufficiently demanding to elicit robust performance differences. These ceiling effects likely reduced behavioral variability and limited sensitivity to subtle impairments in inhibitory control, as well as to potential brain–behavior relationships. In addition, the application of conservative bootstrapping procedures, while increasing the robustness of our statistical inferences, attenuated the significance of several effects, suggesting limited power to detect small-to-moderate group differences in this moderately sized clinical sample. The high variability in our stroke sample additionally complicated interpretation of results, as there can be many variables including network disruption, lesion size, and even proximity to frontal regions that could potentially contribute to observed decreases in FMΘ or the lack of significant behavioral results.

Together, near-ceiling task performance and high variability in lesions may have contributed to the absence of statistically robust behavioral effects and to the marginal nature of some neural findings. Accordingly, at this time we can only provide preliminary information about the impact of stroke on both frontal theta and future studies should therefore combine more challenging or adaptive task designs with larger samples to improve sensitivity and clarify the relationship between FMΘ modulation, behavioral performance, and recovery-related processes.

While individual FMΘ sources could not be localized reliably in our sample, this suggests that stimulation protocols may be better guided by group-level source information derived from healthy controls rather than from unstable individual estimates for the time being. By showing that stroke-related alterations are quantitative rather than categorical, our results suggest that stimulation approaches aiming to upregulate FMΘ could be guided by group-level source information obtained in healthy participants rather than assuming fundamentally different neural dynamics in patient populations. Future work could extend this approach by combining group-derived sources with individualized e-field modelling, thereby enabling stimulation protocols that are both physiologically grounded and tailored to the anatomical variability of patients.

In sum, we demonstrate that task-induced frontal midline theta modulation is attenuated in chronic stroke patients compared with healthy older adults. In healthy older adults, MEG source analysis localized theta modulation to medial frontal regions, consistent with prior MEG/EEG-MEG work implicating medial prefrontal and anterior cingulate generators of FMθ (Iramina et al., 1996, Ishii et al., 2014, Rhodes et al., 2023). Source localization in the stroke group was not robust across the full sample, which we treat as a limitation and a key direction for future methodological work.

The findings highlight FMΘ as a promising but variable target for interventions aiming to enhance cognitive control in stroke rehabilitation. However, our analyses also revealed that reliable individual localization was not achievable with the present approach. This suggests that group-level profiles may offer a more feasible starting point for defining stimulation targets, whereas true individualization will likely require complementary strategies such as electric field modeling. By demonstrating methodological feasibility and outlining key gaps, our results set the stage for future work linking neural oscillations, behavior, and stimulation in clinical populations.

## Declaration of Generative AI and AI-assisted technologies in the writing process

During the preparation of this work the author(s) used ChatGPT 5 in order to debug MATLAB code and improve code efficiency as well as improve readability of sentences. After using this tool, the author(s) reviewed and edited the content as needed and take(s) full responsibility for the content of the published article.

## CRediT authorship contribution statement

**Rebekah I. Brückner:** Writing – original draft, Visualization, Investigation, Formal analysis, Data curation. **Jale Özyurt:** Writing – review & editing, Supervision, Project administration, Investigation, Conceptualization. **Christiane M. Thiel:** Writing – review & editing, Funding acquisition, Conceptualization. **Christoph S. Herrmann:** Writing – review & editing, Conceptualization. **Florian H. Kasten:** Writing – review & editing, Supervision, Data curation, Conceptualization.

## Declaration of Competing Interest

The authors declare that they have no known competing financial interests or personal relationships that could have appeared to influence the work reported in this paper.

## Data Availability

Data will be made available on request.
