# Supplementary figures and images for: Reduced task-induced frontal midline theta activity in chronic stroke patients compared to healthy older adults – An MEG study
Source: Neuroimage Clin. 2026 Mar 6;50:103984. doi: 10.1016/j.nicl.2026.103984 (PMC12997227; doi:10.1016/j.nicl.2026.103984)

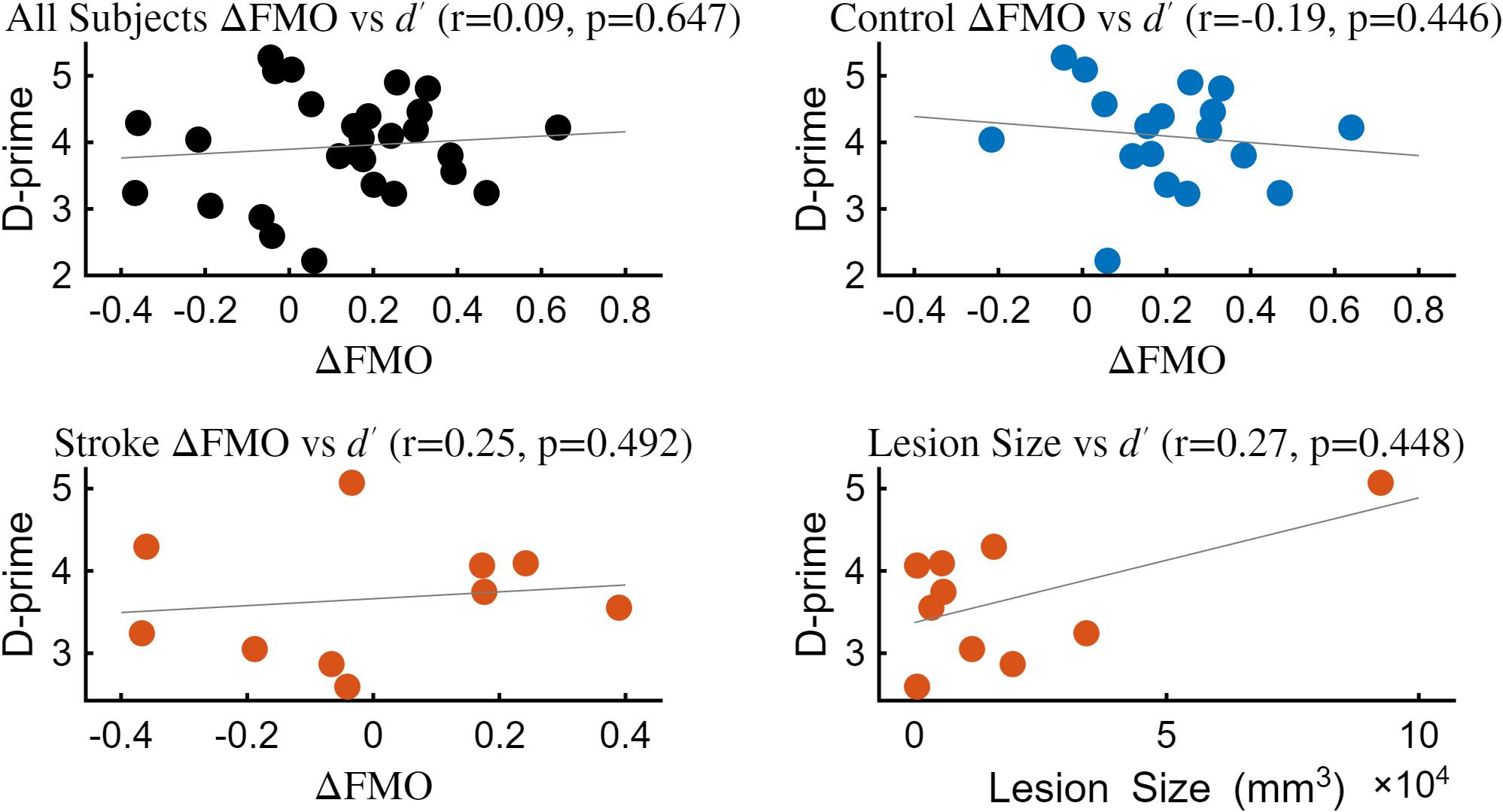

Supplement: Supplementary Fig. 1 [file mmc5.jpg]

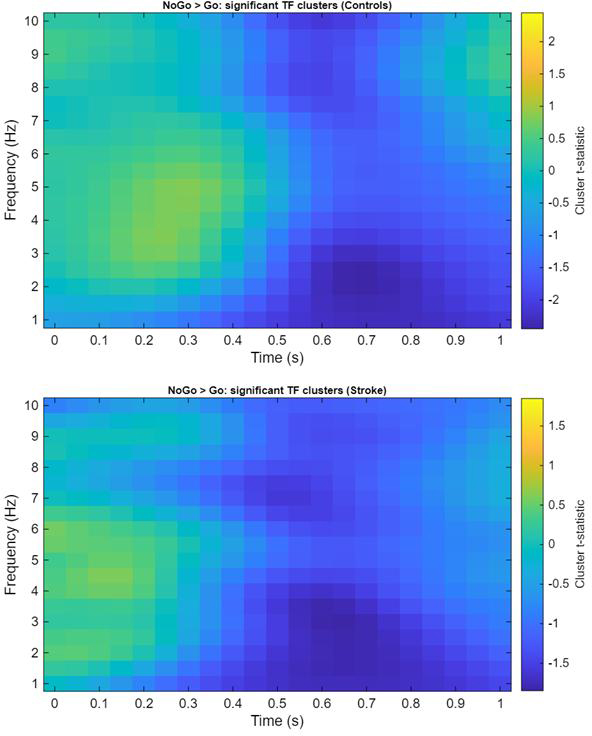

Supplement: Supplementary Fig. 2 [file mmc6.jpg]

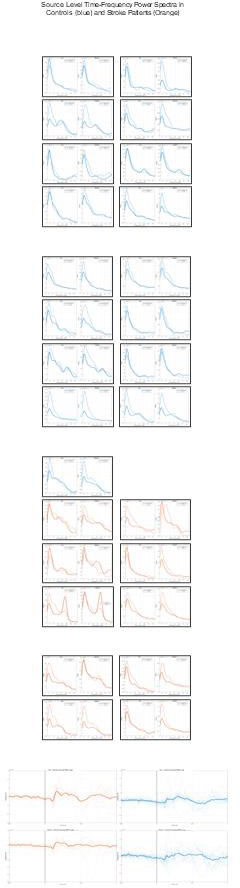

Supplement: Supplementary Fig. 3 [file mmc7.jpg]

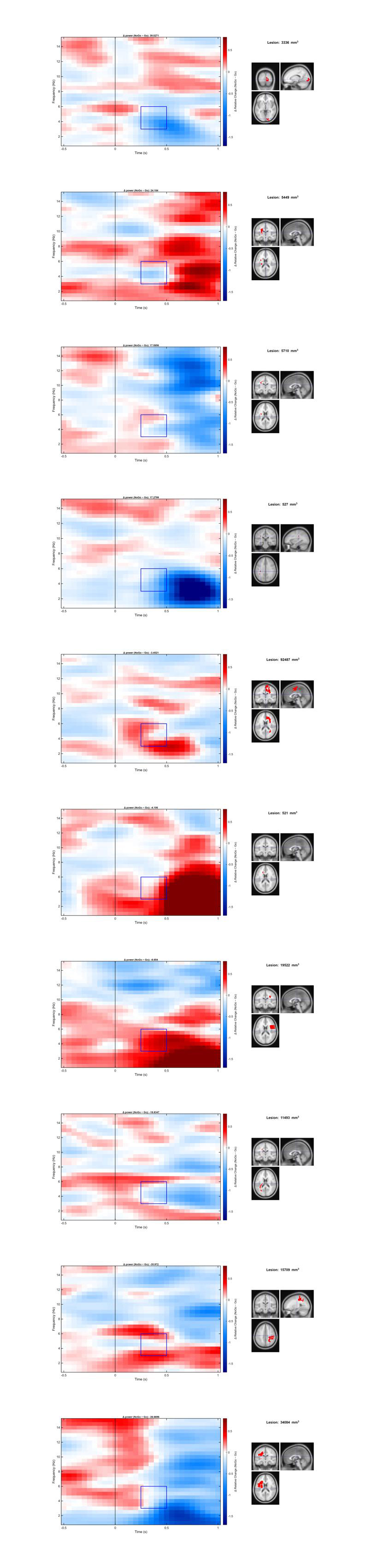

Supplement: Supplementary Fig. 4 [file mmc8.jpg]
