# Supplementary material for: Reduced task-induced frontal midline theta activity in chronic stroke patients compared to healthy older adults – An MEG study
Source: Neuroimage Clin. 2026 Mar 6;50:103984. doi: 10.1016/j.nicl.2026.103984 (PMC12997227; doi:10.1016/j.nicl.2026.103984)
Supplement: Supplementary Data 1 [file mmc1.docx]

Figure S1. *Spearman correlations between all subject’s Δ FMΘ and d‘, controls’ Δ FMΘ and d’, stroke patients’ Δ FMΘ and d’, and lesion size (mm3) and d’.* Spearman’s rank correlation coefficients were computed to assess monotonic relationships. Lines indicate least-squares regression fits for visualization. None of the tests produced significant results. Healthy older controls are mapped in blue and stroke patients are mapped in orange to improve visibility, while combined groups have been plotted in black for distinction.
