# Supplementary material for: Reduced task-induced frontal midline theta activity in chronic stroke patients compared to healthy older adults – An MEG study
Source: Neuroimage Clin. 2026 Mar 6;50:103984. doi: 10.1016/j.nicl.2026.103984 (PMC12997227; doi:10.1016/j.nicl.2026.103984)
Supplement: Supplementary Data 2 [file mmc2.docx]

Figure S2. *Time–frequency t-maps resulting from cluster permutation tests.* Time–frequency maps depict the averaged cluster t-statistics for significant NoGo > Go effects in healthy controls (top) and stroke patients (bottom), obtained from cluster-based permutation testing of source-level data (1–10 Hz, 0–1 s post-stimulus). In controls, a pronounced early theta-band effect (approximately 3–6 Hz, 0.1–0.4 s) is evident, reflecting robust inhibition-related activity. In contrast, stroke patients show weaker and more temporally and spectrally diffuse modulation, indicating reduced consistency of theta dynamics during response inhibition. Color values represent cluster-level t-statistics. No significant clusters could be identified.
