# Supplementary material for: Reduced task-induced frontal midline theta activity in chronic stroke patients compared to healthy older adults – An MEG study
Source: Neuroimage Clin. 2026 Mar 6;50:103984. doi: 10.1016/j.nicl.2026.103984 (PMC12997227; doi:10.1016/j.nicl.2026.103984)
Supplement: Supplementary Data 3 [file mmc3.docx]

Figure S3. *Individual power & frequency plots for all participants and ERFs by condition.* For each subject, we extracted source-level spectral power from the precomputed Go and NoGo time–frequency representations and averaged power across all grid points within the significant cluster ROI and across time within the baseline (−1500 to −500 ms) and active (250 to 500 ms) windows. The resulting ROI-averaged power spectra (1–15 Hz) are shown for baseline (solid) and active (dashed) periods. Virtual channels reflecting the cluster-defined ROI and time windows were selected to match the primary statistical analyses. In addition, event-related fields from averaged frontal MEG channels are displayed for all individuals in butterfly plots, with controls shown in blue and stroke participants in orange. These plots were included as a visualization aid to illustrate inter-individual variability across participants, groups, and conditions.
