# Supplementary material for: Reduced task-induced frontal midline theta activity in chronic stroke patients compared to healthy older adults – An MEG study
Source: Neuroimage Clin. 2026 Mar 6;50:103984. doi: 10.1016/j.nicl.2026.103984 (PMC12997227; doi:10.1016/j.nicl.2026.103984)
Supplement: Supplementary Data 4 [file mmc4.docx]

Figure S4. *Time–frequency representation of relative power differences between NoGo and Go trials (NoGo–Go) for stroke patients.* The left panel shows baseline-corrected changes in oscillatory power across time (−0.5 to 1 s) and frequency (1–15 Hz), with red indicating increased power during NoGo trials and blue indicating decreased power. The black vertical line marks stimulus onset (0 s). The blue rectangle highlights the predefined theta time–frequency window used for subsequent analyses. The right panel displays the corresponding lesion mask (red) overlaid on MNI anatomical images in three orthogonal views, with total lesion volume indicated above (mm³). This example illustrates reduced task-related frontal midline theta modulation in the presence of a focal lesion.
